# A three-year review of lung cancer patient characteristics in a tertiary hospital

**DOI:** 10.4314/gmj.v57i3.2

**Published:** 2023-09

**Authors:** Jane S Afriyie-Mensah, Ernest Kwarteng, John Tetteh, Hafi Gbadamosi, Mary-Ann Dadzie, Yaw Boakye Mensah, Ekow Entsua-Mensah

**Affiliations:** 1 Department of Medicine and Therapeutics, University of Ghana Medical School, Legon, Accra, Ghana; 2 Research Department of the University of Ghana Medical School, Accra, Ghana; 3 Department of Community Health, University of Ghana Medical School, College of Health Sciences, University of Ghana, Accra, Ghana; 4 National Radiotherapy Oncology and Nuclear Medicine Centre, Korle-Bu Teaching Hospital, Korle-Bu, Accra, Ghana; 5 Department of Radiology, University of Ghana Medical School, Legon, Accra, Ghana; 6 National Cardiothoracic Centre, Korle-Bu Teaching Hospital, Korle-Bu, Accra, Ghana

**Keywords:** Lung Cancer, Anaplastic Lymphoma Kinase (ALK), Epidermal growth factor receptor (EGFR)

## Abstract

**Objective:**

The study sought to determine clinical characteristics and histologic subtypes of a cohort of lung cancer patients in a tertiary facility.

**Design:**

Retrospective review of the medical records of histology-confirmed lung cancer cases at the respiratory clinic over a 3-year period.

**Setting:**

Respiratory Clinic, Korle-Bu Teaching Hospital, Accra, Ghana

**Participants:**

All adult patients with histologically diagnosed lung cancer were enrolled.

**Main outcome measures:**

Lung cancer histological types

**Results:**

The proportion of lung cancer cases was 12.4%. The majority were women (57.8%) and the mean age at diagnosis was 55.8±16.0 years. The patients were predominantly non-smokers (61%). Common symptoms were chronic cough and chest pain. More than two-thirds of the cases presented in clinical stages III and IV with the predominant histological subtype being adenocarcinoma in smokers and non-smokers. Genetic testing for epidermal growth factor receptor (EGFR) and Anaplastic Lymphoma kinase (ALK) mutations were largely absent.

**Conclusions:**

The majority of lung cancer patients presented late with advanced disease. Adenocarcinoma was the predominant histological subtype in a predominantly non-smoking population, with an increased prevalence among women less than 60 years. This should encourage testing for genetic mutations to improve patient survival.

**Funding:**

None declared

## Introduction

Lung cancer (lung ca) has become a disease of public health concern, having risen to be the leading cause of cancer mortality in both men and women worldwide, surpassing prostate and breast cancer-associated mortality.^1^,^4^ According to WHO, in 2020, lung cancer was the second most commonly occurring cancer globally, with an estimated 2.21 million new cases.^5^ Although second to breast cancer, it remained the most common cause of cancer mortality, with 1.8 million deaths.^5^ The disease is associated with a significantly poor prognosis, as more than half of the patients die within one year of diagnosis.^6^

This is largely due to late presentation with advanced/metastatic disease. The five-year survival rate of lung cancer varies with the stage of the disease and is generally the lowest among leading cancers.^7^ In a study by Melling et al.,^8^ only about 50% of lung ca patients present with typical symptoms of chronic cough, chest pain, breathlessness, or haemoptysis, whiles the remaining 50% usually present with unsuspecting systemic symptoms resulting in missed diagnosis by primary clinicians hence the late diagnosis with advanced disease.

Tedious referral pathways with prolonged waiting times for specialist respiratory physicians are a significant factor in the diagnostic delays in lung cancer. This could be more daunting in poorly resourced countries.

Securing the diagnosis in suspected patients is usually associated with significant cost implications due to the extensive investigations required. The initial evaluation aims to obtain radiologic and clinical information to guide specific tissue biopsy, staging and management as early as possible. The investigations could include a chest CT scan with contrast, MRI of the chest, Positron emission tomography (PET) scan, and biopsy of suspected lung growth/metastatic tissue via percutaneous route or bronchoscopy. The obtained tissue is subject to histology and/or cytology for definitive diagnosis. Even in advanced healthcare facilities, these investigations can deter patients and families, particularly those without healthcare insurance.^9^ The above cost implications underlie the lack of adequate histological data from most African countries. Lung cancer management is changing fast and depends on disease staging, histology types, and histological subtypes. This has led to the development of targeted therapies in lung ca with better patient outcomes. The range of therapeutic approaches in lung cancer includes chemotherapy, radiotherapy, surgical resection or lung tumour ablation. Multimodal treatment approaches combining different approaches such as chemoradiation or chemotherapy and surgery are often employed depending on disease staging.

Lung cancer rates and mortality trends also vary substantially by sex, age, race/ethnicity, socioeconomic status and geographical location mainly because of the differences in smoking patterns.^10^ With tobacco smoking being the strongest risk factor for lung cancer, it is so that countries with historically significant smoking uptake, such as North America and Europe, record higher numbers of the disease.^3^,^10^ However, lung cancer rates are rising in countries with a rather late increase in tobacco smoking, such as low- and middle-income countries, reported accounting for an alarming 58% of total lung cancer cases globally.^11^,^12^ It has been predicted that the disease burden could shift in the 21^st^ century from the Western world to such countries.^12^ Lung cancer has been shown to occur more frequently in men compared to women, with an incidence of 57.8/100,000 versus 45.9/100,000 respectively in 2015.^7^ However, recent trend analysis depicted a decline in the incidence rates in men whiles that in women appears to have plateaued.^7^ This pictorial change has been attributed partly to more women taking up smoking habits in recent years.^10^ In contrast, there is evidence of an increasing incidence of lung cancer among non-smokers, particularly women, drawing significant scientific interest.^13^

Although low- and middle-income countries are estimated to significantly contribute to global statistics of morbidity and mortality, reports on lung cancer incidence rates in these regions, particularly sub-Saharan Africa, have been scanty compared to that of the developed world.^11^,^14^,^15^ While many African countries perceive a low burden of cancers, there is still a high record of cancer-associated mortality.^14^ GLOBOCAN reports lung cancer as the fourth most common cancer among men in Africa, with 39,300 new cases.^15^ In Ghana, GLOBOCAN statistics in 2020 ranked lung cancer as the 8^th^ most prevalent cancer, being 2.2% of all new cancer cases and listed among the top 10 causes of cancer mortality.^6^ In 2012, Laryea et al. 17 reported lung cancer as the third most common cancer among Ghanaian men, with a prevalence of 5.3%. In Nigeria, lung cancer is ranked 14^th^, with a prevalence of 1.4%.^18^ Disease prevalence may be underestimated in most African countries due to the prevailing significant diagnostic challenges and misdiagnosis of most lung conditions as pulmonary tuberculosis, coupled with low acceptance of post-mortem practices.^19^

There is a pressing need to determine the prevalence and clinical characteristics of patients diagnosed with lung cancer in the sub-region. Obtaining this information will help create awareness among clinicians and improve diagnostic pathways for suspected lung cancer cases. It will also lead to the development of beneficial policies in lung cancer management. The current study retrospectively reviewed the clinical and histological characteristics of patients diagnosed with lung cancer at the respiratory clinic of the Korle-Bu Teaching Hospital, Accra-Ghana. This review is expected to be a springboard for future prospective studies.

## Methods

This retrospective study involved the review of the medical records of lung cancer cases diagnosed (histopathology/cytology confirmed) at the respiratory clinic of the Korle-Bu Teaching Hospital from January 2016 to December 2018 (n = 90). Korle-Bu Teaching Hospital is the premier tertiary hospital in the country and is endowed with many specialist clinics and centres of excellence. It is thus a major referral facility serving the Greater Accra region, the entire nation, and some neighbouring countries. The respiratory clinic, a clinical care unit under the Department of Internal Medicine and Therapeutics, has practising pulmonologists who provide specialist services for various respiratory diseases. The clinic runs weekly on an outpatient basis and attended to 721 new cases over the study period.

### Data Collection

Patients' demographic characteristics and clinical information were obtained from the clinic's medical records with the help of an abstraction tool and were given unique codes for confidentiality. Demographic data collected included age, gender and occupational history, whereas clinical information included presenting symptoms and their respective duration of onset, smoking history, clinical cancer stage using the Tumour, Nodes and Metastasis (TNM)-7 clinical staging for lung cancer 20, mode of investigation used to obtain biopsy for histological diagnosis, histologic subtype of lung cancer, and specific mutations detected such as Epidermal growth factor receptor (EGFR) and Anaplastic lymphoma kinase (ALK) mutations. The presence of histopathology or cytology reports in the medical notes was necessary to objectively determine the type of lung cancer and the histological subtype.

For the study, active smokers were defined as those who directly smoked cigarettes (direct inhalation from a cigarette) at some point in their lives, either at the time of presentation or as an ex-smoker. A passive smoker was one with a documented history of having lived/living in close contact with an “every day” smoker (spouse, parent, sibling or co-worker) and indirectly exposed to cigarette smoke for any duration in a given day (CDC, 2006). An “every day” or regular smoker is defined as one who smokes every day or smoked more than 100 cigarettes in their lifetime (CDC, 2006).

### Data Analysis

The data obtained was collated, cleaned with Microsoft Excel, and exported to Stata 16.1 for data cleaning and analysis. Descriptive analysis was performed to summarise socio-demographic characteristics. The chi-square test assessed differences in histological proportion by smoking status, and significance was set at p<0.05.

Ethical approval was obtained from the Ethical and Protocol Review Committee of the College of Health Sciences, University of Ghana (Protocol identification number – CHS-Et/M.5-4.17/2020-2021).

## Results

### Patient characteristics

A total of 90 cases (12.4%) of all the new cases seen at the respiratory clinic over the period were diagnosed with lung ca as documented in the medical records. However, histopathology or cytology reports to confirm diagnosis were found in 85 of them. The mean age of the patients was 55.8±16.0 years, with 39(44%) of them being 60 and above, 35 (38.9%) being between 40 and 59, and 16 (17.8%) being less than 40 years. Most (58%) of the cases were females, and 63.5% were diagnosed below age 60, compared to 47.4% of males below age 60. Regarding smoking, about 61% were non-smokers, 16.7% were active smokers, and 6.7% were considered passive smokers. The socio-demographic characteristics of the patients are summarised in Table 1.

**Table 1:** Socio-demographic characteristics of patients in the study (n=90)

Variable	n(%)
**Sex**	
**Female**	52(57.8)
**Male**	38(42.2)
**Age group**	
**≤39**	16(17.8)
**40-59**	35(38.9)
**≥ 60+**	39(43.3)
**Mean age = 55.8±16.0 years**	
**Males < 60**	18(47.4)
**Males ≥ 60**	20(52.6)
**Females < 60**	33(63.5)
**Females ≥ 60**	19(36.5)
**Occupation**	
**Teachers**	17(18.9)
**Unemployed**	5(5.6)
**Trading**	17(18.9)
**Artisan**	13(14.4)
**Industrial**	4(4.4)
**Administrative**	17(18.9)
**Unskilled**	10(11.1)
**Nursing**	5(5.6)
**Farming**	2(2.2)
**Smoking status**	
**Active**	15(16.7)
**Passive**	6(6.7)
**Non-smoker**	55(61.1)
**Missing**	14(15.6)

As shown in Table 2, 53/82 (64.6%) had a chronic cough, with about 96% reporting a duration of at least a month. Haemoptysis was reported by 10/83(12.0%), all lasting at least a month. About 40% of the cases had chest pain that had persisted for at least a month in about 94%. Breathlessness was reported by 28.0% of the patients, with a minimum duration of a month in the majority. Additional symptoms such as weight loss, anorexia, shoulder pain and fatigue were sparsely documented. Clinical staging of lung cancer was documented in 64/90 (71.1%) of the cases, and 56/64(87.5%) were in clinical Stage IV, 6/64(9.4%) in clinical stage III and 2/64(3.1%) in stage II.

**Table 2:** Clinical presentation and clinical staging of patients in the study (n=90)

Variable	n(%)
**Cough history**	
**No**	29(32.2)
**< 1 month**	2(2.2)
**1-3 months**	16(17.8)
**≥ 4 months**	35(38.9)
**Missing**	8(8.9)
**Haemoptysis**	
**No**	73(7.8)
**1-3 months**	3(81.1)
**≥ 4 months**	7(3.3)
**Missing**	7(7.8)
**Chest pain**	
**No**	49(54.4)
**< 1 month**	2(2.2)
**1-3 months**	9(10.0)
**≥ 4 months**	22(24.4)
**Missing**	8
**Breathlessness**	
**No**	59(65.5)
**< 1 month**	2(2.2)
**1-3 months**	13(14.4)
**≥ 4 months**	8(8.9)
**Missing**	8(8.9)
**Clinical Staging**	
**Missing**	26(28.9)
**IIa**	1(1.1)
**IIb**	1(1.1)
**IIIa**	3(3.3)
**IIIb**	1(1.1)
**IIIc**	2(2.2)
**IVa**	38(42.2)
**IVb**	18(20.0)

### Histological Diagnosis and Classification

The mode of obtaining tissue for histopathology was via image-guided percutaneous lung biopsy in 57/83(68.7%) whiles 21/83(25.3%) had bronchoscopy with endobronchial biopsy. For histological diagnoses, 75/85(88.2%) had non-small cell lung cancer (NSCLC). The subtypes of NSCLC comprised mainly of adenocarcinoma 59/75(78.7%), Squamous cell carcinoma 9/75(12%) and 4/75(5.3%) had undifferentiated NSCLC.

Other subtypes of NSCLC in the minority were adenosquamous carcinoma (1/75), lymphoepithelioma-like carcinoma (1/75) and large cell carcinoma (1/75). Bronchial carcinoid tumours occurred in 6/85(7.1%), and only 2/85(2.4%) had small cell lung cancer (SCLC). Evidence of testing for mutations such as EGFR and ALK was predominantly absent, with 3 EGFR and 4 ALK results documented. The details are presented in Table 3.

**Table 3:** Investigations carried out for patients in the study (n=90)

Variable	n(%)
**Investigation**	
**Biopsy (Suprarenal mass)**	1(1.1)
**Bronchoscopy**	21(23.3)
**Image-guided biopsy**	59(65.6)
**Pleural aspirate**	4(4.4)
**Missing**	5(5.6)
**Histology**	
**Missing**	5(5.6)
**Atypical Adenomatous Hyperplasia (AAH)**	2(2.2)
**Non-small cell lung ca (NSCLC)**	75(83.3)
***Adenocarcinoma***	59(65.6)
***Adenosquamous***	1(1.1)
***Lymphoepithelioma-like carcinoma***	1(1.1)
***Squamous cell carcinoma***	9(10.0)
***Undifferentiated lung CA***	4(4.4)
***Large cell carcinoma***	1(1.1)
**Bronchial carcinoid tumour**	6(6.7)
**Small cell Lung carcinoma (SCLC)**	2(2.2)
**eGFR**	
**Missing**	86(95.6)
**Negative**	2(2.2)
**Positive**	2(2.2)
**ALK**	
**Missing**	87(96.7)
**Not Done**	1(1.1)
**Positive**	2(2.2)

Approximately 39.5% of the males were active smokers, whiles about 9.6% of the females were passive smokers. Adenocarcinoma was the predominant subtype irrespective of smoking status. Of the 9 cases with squamous cell ca, 6(66.7%) were non-smokers. There was a significant association between sex, histological subtypes and smoking status (Table 4).

**Table 4:** Relationship between sex, lung Ca histology and smoking habits among patients in the study (n=90)

Variable	Smoking status				
	Active	Passive	Non-smoker	Missing	*p*-value
	n(%)	n(%)	n(%)	n(%)	
**Sex**					
**Female**	0(0.00)	5(9.62)	38(73.08)	9(17.31)	**<0.001** *
**Male**	15(39.47)	1(2.63)	17(44.74)	5(13.16)	
**Histology sub-types**					
**AAH**	1(50.00)	0(0.00)	0(0.00)	1(50.00)	**0.001** *
**Adenocarcinoma**	9(14.29)	4(6.35)	45(71.43)	5(7.94)	
**Adenosquamous**	0(0.00)	1(100)	0(0.00)	0(0.00)	
**Lymphoepithelioma-like carcinoma**	0(0.00)	0(0.00)	1(100)	0(0.00)	
**Large Cell carcinoma**	1(100)	0(0.00)	0(5.45)	0(0.00)	
**Squamous cell carcinoma**	2(22.22)	0(0.00)	6(66.67)	1(11.11)	
**SCLC**	1(50.00)	0(0.00)	0(0.00)	1(50.00)	
**Bronchial carcinoid**	0(0.00)	1(16.67)	3(50.0)	2(22.22)	

* statistically significant (*p*< 0.05), test: Chi-square test.

## Discussion

The estimated prevalence of lung cancer in Ghana by Laryea et al., 17 in 2012, was 5.3%, but anecdotal evidence suggests this could be higher than estimated ten years ago. The recent increase in lung cancer diagnosis may be attributed to improved diagnostic equipment and techniques such as the chest CT scan with skill in image-guided lung biopsies, flexible bronchoscopy and access to some well-resourced histopathology laboratories.

This allows us to carry out prospective studies in lung cancer to fill the existing gap in data. The current review, however, presents important baseline information on patient clinical characteristics, diagnostic approaches and histological types of lung cancer seen in our setting.

The mean age of the lung cancer patients was 56 + 16 years, with the majority being women (58%). The typical age of lung cancer diagnosis is 65 years and over, with an average of 70 years.^21^ On the contrary, the proportion of patients in our cohort aged 60 and above was comparable to those in the 40–59-year group. According to the data from the National Cancer Institute in the US, the estimated percentage of lung cancer patients under age 45 was 5.2% or below.^22^ In contrast, about 18% of our lung cancer cases were below age 40, depicting a relatively younger age of onset of the disease among the reviewed cases.

Lung cancer among women has been increasing worldwide, notably among female never-smokers and has been described as a modern epidemic. 23-25 Some studies have shown that women are more likely to develop lung cancer at younger ages (30-54 years) compared to men. 26 While only about 20% of men with lung cancer are non-smokers, as much as 50% of women with the disease are non-smokers.^13^ We observed a similar trend in this review, with women not only being in the majority but about 64% were diagnosed with lung cancer below age 60. Increasing lung cancer rates in women have been historically attributed to increasing smoking habits among women in the Western world. Still, current studies have shown an increasing trend, even without smoking. This picture was similarly observed in the current review, where all the women were never smokers, with only a handful (five) having been passively exposed to smoking. A similar study in Eastern and Southern Asia found that 83% of females diagnosed with lung cancer were non-smokers.^24^,^27^ In the absence of tobacco smoking among these women, some studies have suggested a link between female sex hormones, particularly oestrogen, and increased lung cancer risk. In fact, some laboratory studies have shown that oestrogen does promote lung cancer progression.^25^,^28^,^29^

Exposure to biomass fuels for cooking, predominantly practised in developing countries and Asia, has been thought to be a significant etiological risk factor for lung ca in non-smoking women in these regions.^30^, ^31^ In a meta-analysis by Kurmi et al^32^ pooled data estimated the risk of lung cancer to be 70% among solid fuel users compared to those without the exposure.

In Sub-Saharan Africa, about 80% of the population relies on biomass fuels for cooking (wood, charcoal, crop residues/dung and coal) with minimal use of LPG gas.^33^ However, Data on its influence on lung cancer development is scanty. Smoke from burning household waste, a duty mostly performed by women, has been associated with an increased risk of lung cancer as it increases environmental particulate matter and dioxins. 34, 35 Other generally identified risk factors of lung ca include genetic predisposition, indoor or workplace exposure to radon gas and other chemicals, family history of lung cancer and previous radiation therapy to the chest.^30^

Although the current review could not assess biomass fuel exposure and exposure to other risk factors, the predominant occupation being teachers, traders and administrative officers suggests an almost insignificant exposure to workplace substances or gases among the patients. The supposedly risky occupations, such as industrial work and farming, were minimal among the studied cohort. A prospective study to better quantify exposure to these factors and assess risk will be pertinent to provide more baseline information for health interventions.

Lung cancer could be asymptomatic in the early stages; however, the common presenting symptoms include cough, chest pain, haemoptysis and breathlessness.^36^ Cough is the most common symptom occurring in 5075% of lung ca patients, and this was similarly noted in 51% of the current cases reviewed.^36^ Chest pain and breathlessness were reported by 40% and 28% of the patients, respectively, as similarly observed in a study by Kocher et al.^37^ Typically, haemoptysis is a less prevalent symptom in lung ca. Chute et al.,^38^ reported a 27% prevalence of haemoptysis among study participants, but the current study showed a much lower prevalence of 12% among the cases. From the results, the duration of patients' symptoms before reporting was averagely between one to three months, depicting a late presentation to the specialist clinic. This could be attributed to poor health-seeking behaviours, misdiagnosis as pulmonary tuberculosis or late referral from initial health facilities.^8^,^19^,^39^ Misdiagnosis of lung ca as pulmonary tuberculosis appears significant in most African countries where TB is endemic, obscuring the true numbers of other lung diseases such as lung cancer.^40^ Anecdotal evidence in our setting shows that many lung cancer cases would have completed or been initiated on PTB treatment before their referral. Thus, it is unsurprising that the predominant clinical staging of the disease at presentation was stage IV (advanced disease) in close to 88% of the study cohort, which only casts a poor shadow on patient survival curves.

Traditionally, NSCLCs account for about 80% of all lung cancers, and this was confirmed in the current review, with 83% having NSCLC. 41

Adenocarcinoma forms the predominant histological subtype of NSCLC, accounting for close to 66% of all NCSLC in this study cohort, followed by 10% squamous cell cancer subtype. 41 Adenocarcinomas are also known to be the most prevalent subtype of lung ca among non-smokers 41, as found in about 71% of non-smokers in the current study. The prevalence of SCLC has been dwindling due to a decline in smoking habits globally.^42^ Comparatively, we also found a low prevalence of SCLC (2.2%) among the study cohort. Bronchial carcinoid tumours, which are relatively rare, were found in about 7% of the patients.

The presence of mutations in adenocarcinoma histological subtypes such as EGFR, ALK and PDL1 has contributed to developing novel and more tolerable anti-cancer therapies, with improved survival in the otherwise poor prognosis associated with these tumours.^43^ This review shows that only a negligible proportion (5 patients) had evidence of requested mutations with two positive results for EGFR and ALK mutations, respectively. Although there might be physician willingness to assay for these mutations, the major limitations are the lack of in-country testing facilities and prohibitive costs where available. The lack of genetic testing prevents these patients from benefiting fully from the novel targeted therapies available and could definitely impact patient survival.^44^ Efforts should be made by the relevant stakeholders in the country to provide affordable testing for these lung cancer mutations.

### Study Limitations

Being a retrospective study, we relied on manually archived medical records. We, therefore, encountered challenges such as missing information, illegible clinical notes or simply absent needed information. These could have affected study results and conclusions.

## Conclusion

The majority of the lung cancer patients presented late with advanced disease. The study provides objective evidence that adenocarcinoma is the predominant histological subtype in a predominantly non-smoking population, with an increased prevalence among women less than 60 years. This should encourage testing for genetic mutations to improve survival.
